# Medical Association of Georgia

**Published:** 1897-02

**Authors:** Geo. H. Noble

**Affiliations:** President; Atlanta, Ga.


### CORRESPONDENCE.

### MEDICAL ASSOCIATION OF GEORGIA.

ATLANTA, GA., January 1, 1897.

MY DEAR DOCTOR: No pains will be spared in the efforts to make the coming meeting of the Medical Association of Georgia the largest, the most interesting and the most profitable meeting in the history of the Association. A number of the most prominent members of the profession, representing the different departments of medicine and surgery, will be present from various parts of the country, adding much scientific interest and value to the proceedings.

The Association has reached a point where members cannot afford to remain away from its meetings without detriment to themselves professionally. The high character of its work is expressed in a letter from the able and delightful pen of Dr. Samuel Lloyd, of New York, published in the *New York Post Graduate Journal.* He says: "While the atmosphere of the meeting (1896) was distinctly scientific, there was also a marked tendency to have a good time. Looking back at the meeting one cannot but feel that the average of the work was of a most superior character. It compares favorably with any meeting of the New York State Medical Society that I have attended, and there can be no question that, aside from the rest one gets by a trip to these State Societies, *a great deal of information may be gathered if the rest of the State Societies are of an equally high average as that of the State of Georgia.*"

Now that political and other disagreeable features have been removed from the meetings and a great impetus given to the furtherance of scientific, practical medicine and surgery, it is hoped that each member entertains an equally high appreciation of the good work of the Association as others outside the State, and that all will perform the duties encumbent upon them as members of a scientific body. Therefore, I personally appeal to you to make an earnest effort to be present and contribute to the success of the coming meeting in April next, at Macon, Ga.

Most respectfully yours,

GEO. H. NOBLE, President.

[We presume all members of the Association have received copies of the above letter from the President, Dr. Noble. THE JOURNAL takes pleasure in adding its appeal to members to be present at the Macon meeting and assist in the upbuilding and strengthening of our State Association. This Association ought to be the pride of each individual member, and each should be glad to contribute his energies and influence to make it a success. We are informed by Dr. Noble that there are already indications pointing to a good meeting in April, and our Macon friends say that preparations are making for a "red-hot" time, whatever that may mean. Get ready, Doctor, write a good paper and don't fail to be on hand at the proper time.—ED.]

